# Supplementary material for: Identifying Behavior Change Techniques Used in Tobacco Cessation Interventions by Oral Health Professionals and Their Relation to Intervention Effects—A Review of the Scientific Literature
Source: Int J Environ Res Public Health. 2021 Jul 13;18(14):7481. doi: 10.3390/ijerph18147481 (PMC8305605; doi:10.3390/ijerph18147481)
Supplement: Supplementary file 1 [file ijerph-18-07481-s001.zip › ijerph-1284272-supplementary.pdf]

Table S1. Characteristics of the included studies.

#	Authors, year	Design	Participants	Intervention	Comparison	Intervention effect
1	Gordon, J. S. et al., 2005, USA Ref * [18]	Quasi-experimental time-series design	364 tobacco- users, mean age 38.6 years 58% men	Five A's: Ask, Advise, Assess, Assist and Arrange	Usual care	Self-reported tobacco cessation (point prevalence): At 3 months: adjusted OR (95% CI) = 4.85 (1.20, 19.60), $p < 0.05$ At 6 months: OR (95% CI) = 5.25 (1.35, 20.36), p < 0.01
2	Virtanen S. E. et al., 2015, Sweden Ref [19]	Cluster randomized controlled trial	467 tobacco- users, mean age in years 45.57 and 63.4% men	Five A's: Ask, Advise, Assess, Assist and Arrange	Usual care	At 3 months: self-reported abstinence: OR (95% CI): 3 (1.07 to 5.91), $p = 0.03$
3	Walsh M. M et al., 2003 USA Ref [20]	Cluster-randomized controlled trial	307 tobacco users from athletes in 44 high schools	*Peer-led* 50- to 60-min educational team meeting, video tape presentation 10–15 min, small groups discussion on tobacco advertising. *Oral cancer screening*, tailored advice to quit, self-help written material, single tobacco cessation counselling in small groups.	Usual care	At 12 months: self-reported no current use of ST: OR (95% CI) = 2.29 (1.28–4.08). $p < 0.00$
4	Nohlert E. et al., 2009 Sweden Ref [21]	Two arms randomized uncontrolled trial	300 smokers over 20 years, 75% women	High-intensity treatment (HIT): Eight (40 min) individual counseling sessions based on behavior therapy, coaching and pharmacological advice.	Low-intensity treatment (LIT): Single (30 min) focus on contents of traditional self-help program.	At 12 months: self-reported point prevalence abstinence of smoking in the past seven days: OR (95%) = 1.6 (0.9–2.8) $p= 0.11$. Continuous abstinence: not one puff of smoke in the past six months: OR (95% CI) = 2.3 (1.1–4.4) $p= 0.02$
5	Binnie I. V. et al., 2007, UK Ref [22]	Randomized controlled trial-pilot study	116 smokers 59 (intervention), mean age 39.9 years 57 (control), mean age: 43.5 years	Five A's: Ask, Advise, Assess, Assist and Arrange.	Usual care	At 3 months: Self-reported point prevalence: OR (95% CI) = 0.53 (.16 to 1.70). $p = 0.28$ CO ** point prevalence: OR (95% CI) = 0.53 (.16 to 1.70), $p = 0.28$
6	Gordon, J. s et al, 2010, USA Ref [23]	Randomized trial	2549 smokers Mean age: 40.5 years	Five A's: Ask, Advise, Assess, Assist and Arrange.	Usual care	At 7.5 months: self-reported prolonged abstinence: OR (95% CI) = 3 (1.78 to 4.67), $p <.00$
7	Stevens V. J. et al., 1995, USA Ref [24]	Randomized controlled trial	518 male users of tobacco, 15 years and older	Oral examination, advise to quit, describe the harmful effect of tobacco, watch 9-min videotape, set specific quit date, self-help booklet, telephone number of 24-hr advice line, quit kit, follow-up phone call.	Usual care	At 3 months: self-reported point prevalence abstinence in the past 7 days: All tobacco: OR (95% CI) = 2 (1.09 to 2.90), $p = 0.01$
8	Andrews A. J. et al., 1999, USA Ref [25]	Randomized controlled trial	633 ST users, mean age 36 years 239 (control) and 394 (intervention)	Assess tobacco use, oral examination, advice to quit related to oral health, written materials, quit kit, set quit date, motivational video, follow-up phone call.	Usual care	At 12 months: self-reported sustained abstinence: OR (95% CI) = 3 (1.50 to 7.09), $p < 0.00$
9	Severson H. H. et al., 1998, USA	Randomized clinical trial	3603 tobacco users, 15 years and older	*Minimal intervention arm*	Usual care	At 3 months: self-reported tobacco abstinence in the past seven days:

	Ref [26]			Assess tobacco use, identify oral change due to tobacco and point it out to the patient, advise to quit, written materials and quit kit. <u>*Extensive intervention arm*</u> In addition to minimal intervention items, set quit date, motivational video, follow-up call.		Extended intervention vs usual care (all tobacco): OR (95% CI) = 2 (1.23 to 2.14), $p < 0.00$. Minimal intervention vs usual care (smoking only): OR (95% CI) = 1.09 (.76–1.55), $p = 0.64$
10	Cohen J. S. et al., 1989 USA Ref [27]	Randomized controlled trial	1027 smokers, 18–64 years with mean age 37 years	<u>*Gum*</u>: 4 step protocols' booklets; 1- ask about smoking, 2- deliver a firm quit message, 3- mutually agree on a quit date, 4- check on each patient progress regularly and nicotine gum. <u>*Reminders*</u>: 4 step protocols booklets and two stickers to remind dentists to follow the protocol. <u>*Both gum and reminder:*</u> all intervention methods and stickers performed.	Counsel smokers with 4 steps protocol's booklet only	At 6 months: self-reported cessation: Gum vs. control: OR (95% CI) = 3.05 (1.34–6.97), $p < 0.00$.
11	Greene. C. J et al., 1994, USA Ref [28]	Randomized clinical trial	96 baseball players using ST 50(minimum intervention), 46(extended intervention)	<u>*Extended intervention*</u>: ST group cessation counseling, oral examination and photographs of advanced ST lesions and disfigurement in players' mouth, verbal message about harmful effects of ST and self-help materials. 15–20 min individual counseling, NRT and non-tobacco substitutes.	<u>*Minimum intervention:*</u> 5–10 min ST group cessation counseling and oral examination	At 6 months: self-reported abstinence: minimum intervention vs extensive intervention: OR (95% CI) = 0.04 (.00 to 0.69), $P = 0.03$
12	Gonseth S. et al., 2010 India Ref [29]	Repeated measures single pretest-posttest (Pilot study)	39 adult smokers mean age 36 (22–53), 59% women	<u>*Smoking cessation intervention*</u>: 4 sessions of individual counseling + NRT or bupropion. <u>*Dentist intervention*</u>: Oral examination, identification of lesions related to tobacco use, information about effects of smoking on oral health, and a second visit to reinforce the importance and correlation of smoking and periodontitis.	Baseline	At week 8: self-reported abstinence: 17 (44%). At 6 months: self-reported abstinence: 6 (15%)
13	Gansky A. S. et al., 2005, USA Ref [30]	Stratified, cluster-randomized controlled trial	1585 tobacco users' athletes 883(intervention) 702(control)	<u>*Dental component:*</u> oral examination, advise ST users to stop, point out problems associated with ST in players' own mouths, self-help guide, single 10- to 15-minute individual sessions of ST cessation counseling <u>*Athletic trainer:*</u> Group discussion, coping problem solving and support *Peer-led component:* single, interactive, educational team meeting, 5-minute video tailored to baseball athletes.	Usual care	At 12 months: self-reported abstinence: 36% in intervention group and 37% in the control group (GEE OR (95% CI) = 0.94 (0.70–1.27)
14	Severson H. H. et al., 2009, USA Ref [31]	Randomized controlled trial	785 tobacco users of active duty military personnel 392(intervention) 393(control)	ST cessation manual, videotape cessation guide, three 15-min telephone counseling using motivational interviewing.	Usual care	At 3 months: self-reported 7-day point prevalence (all tobacco) abstinence: OR (95% CI) = 4 (2.48 to 5.84) $p < 0.00$

15	Secker-Walker et al., 1988, USA Ref [32]	Repeated measures single pretest-posttest (Pilot study)	51 smokers 63% women mean age 43.4 years	Brief counseling protocol: questionnaire administration, discussion of oral health risks and quitting benefits, firm quit-smoking message, problem solving, printed tip sheets to smokers and their partners.	Baseline	At six months: self-reported abstinence: 14.6% (*n* = 7)

* Ref = Reference number; ** CO= Carbon monoxide measuring in exhaled breath.
